# Draft Genome Sequence of *Erythrobacter vulgaris* Strain O1, a Glycosyl Hydrolase-Producing Bacterium

**DOI:** 10.1128/genomeA.00457-15

**Published:** 2015-05-14

**Authors:** Amira Suriaty Yaakop, Chia Sing Chan, Ummirul Mukminin Kahar, Robson Ee, Kok-Gan Chan, Kian Mau Goh

**Affiliations:** a Faculty of Biosciences and Medical Engineering, Universiti Teknologi Malaysia, Skudai, Johor, Malaysia; b Faculty of Science, Division of Genetics and Molecular Biology, Institute of Biological Sciences, University of Malaya, Kuala Lumpur, Malaysia

## Abstract

*Erythrobacter vulgaris* strain O1, a moderate halophile, was isolated from a beach in Johor, Malaysia. Here, we present the draft genome and suggest potential applications of this bacterium.

## GENOME ANNOUNCEMENT

*Erythrobacter* spp. are marine bacteria that are placed under the class *Alphaproteobacteria*. In culture, the bacterial colonies appear as yellow, orange, or red, and the cells usually tolerate salt concentrations as high as 12% NaCl. They hydrolyze gelatin and urea poorly. *Erythrobacter* spp. are fouling marine bacteria (1), and the antifouling agent isatin has been used to control their growth (2). Several *Erythrobacter* spp. are known to exhibit epoxide hydrolase (epoxide hydratase) activity, and at least three of the recombinant epoxide hydrolase enzymes from *Erythrobacter* spp. have been studied extensively (3). These epoxide hydrolases can be used as detoxification agents during drug metabolism. *Erythrobacter* spp. also produce highly efficient manganese-oxidizing enzymes (4). A protein sequence for a haloalkane dehalogenase (Dh1A) was identified in our sequencing reads, but laboratory testing is needed to determine the ability of the bacteria to degrade xenobiotic compounds. Hu and MacMillan analyzed two natural products formed by *Erythrobacter* spp. (5), erythrazole A and erythrazole B. Erythrazole A was found to possess a benzothiazole moiety, which is very rare in natural products. Erythrazole B was shown to be cytotoxic to non-small-cell lung cancer cell lines.

Genomic DNA from the strain O1, which was isolated from a Malaysian beach, was sequenced on the Illumina MiSeq machine. An average coverage of 166-fold was obtained for the 2,858,586-bp (G+C content 62.4%) genome, in 11 contigs (largest: 971,547 bp, shortest: 658 bp, and *N*_50_ is 522,997 bp). The *de novo* assembly was done with IDBA-UD version 1.0.9 (6), and gene prediction was performed using Prodigal (7). The total coding region is 2,612,034 bp, with 2,777 CDSs, 3 rRNA genes, 50 tRNA genes, and 1 tmRNA gene. The longest protein contains 2,955 residues and has low similarity (40%) to the hemolysin-type calcium-binding repeat protein of *Sphingobium chlorophenolicum*. Additionally, seven CDSs for cyclolysin and several other proteins involved in type I secretion were identified.

Based on previous findings, *Erythrobacter litoralis* does not hydrolyze starch in culture (8), yet its genomic data suggest the presence of two alpha-amylases (9). The sequences of these two homolog alpha-amylases show 84% and 75% identity with those of the corresponding sequences in strain O1. However, these alpha-amylases in strain O1 have not yet been biochemically characterized. An alpha-galactosidase was also found in *E. vulgaris* O1, but not in other genomes of *Erythrobacter* spp. The other interesting enzymes found in these species that have potential industrial application are beta-galactosidase and esterase-lipase. Thus, the draft genome sequence of *E. vulgaris* O1 provides potential opportunities for the industrial application of novel enzymes, which had not been explored until now.

### Nucleotide sequence accession numbers. 

This whole-genome shotgun project has been deposited at the DDBJ/EMBL/GenBank under the accession number CCSI00000000. This version of the project has the accession number CCSI01000000.
